# Effects of Live and Pasteurized Forms of Akkermansia from the Human Gut on Obesity and Metabolic Dysregulation

**DOI:** 10.3390/microorganisms9102039

**Published:** 2021-09-27

**Authors:** Yura Choi, Shambhunath Bose, Jaegu Seo, Joo-Hyun Shin, Dokyung Lee, Yesol Kim, Seung Goo Kang, Hojun Kim

**Affiliations:** 1 Department of Rehabilitation Medicine of Korean Medicine, Dongguk University, 814 Siksa-dong, Ilsandong-gu, Goyang-si 10326, Korea; youla21@naver.com; 2 Department of Bioscience, Sri Sathya Sai University for Human Excellence, Navanihal, Okali Post, Kamalapur, Kalaburagi 585313, Karnataka, India; shambose@yahoo.com; 3 Research & Development Center, Enterobiome Inc., 814 Siksa-dong, Ilsandong-gu, Goyang-si 10326, Korea; jgseo@enterobiome.com (J.S.); jhshin@enterobiome.com (J.-H.S.); dklee@enterobiome.com (D.L.); 4 Division of Biomedical Convergence, College of Biomedical Science, Kangwon National University, Chuncheon 24341, Korea; iamfine@kangwon.ac.kr (Y.K.); sgkang@kangwon.ac.kr (S.G.K.); 5 Institute of Bioscience & Biotechnology, Kangwon National University, Chuncheon 24341, Korea

**Keywords:** *Akkermansia muciniphila*, pasteurized bacteria, live bacteria, metabolic disorder, immune modulation

## Abstract

*Akkermansia muciniphila* (*A. muciniphila*) is a promising probiotic candidate owing to its health-promoting properties. A previous study reported that the pasteurized form of *A. muciniphila* strains isolated from human stool samples had a beneficial impact on high-fat diet-induced obese mice. On the other hand, the differences in the probiotic effects between live and pasteurized *A. muciniphila* on the metabolism and immune system of the host are still inconclusive. This study examines the differences between the live and pasteurized forms of *A. muciniphila* strains on the lipid and glucose metabolism and on regulating the inflammatory immune responses using a HFD-fed obese mouse model. The animals were administered the live and pasteurized forms of two *A. muciniphila* strains five times per week for the entire study period of 12 weeks. Both forms of the bacterial strains improved the HFD-induced obesity and metabolic dysregulation in the mice by preventing body-weight gains after one week. In addition, they cause a decrease in the weights of the major adipose tissues, adipogenesis/lipogenesis and serum TC levels, improvement in glucose homeostasis and suppression of inflammatory insults. Furthermore, these treatments restored the damaged gut architecture and integrity and improved the hepatic structure and function in HFD-induced animals. On the other hand, for both bacterial strains, the pasteurized form was more potent in improving glucose tolerance than the live form. Moreover, specific *A. muciniphila* preparations with either live or pasteurized bacteria decreased the number and population (%) of splenic Treg cells (CD4+ Foxp3+) significantly in the HFD-fed animals, further supporting the anti-inflammatory properties of these bacteria.

## 1. Introduction

Metabolic dysregulation represents a cluster of metabolic abnormalities, such as hyperglycemia, hyperinsulinemia and hyperlipidemia. Therefore, it is a vital measure of obesity-related diseases, such as fatty liver disease, insulin resistance and type 2 diabetes [1]. In addition to systemic inflammation, metabolic dysregulation is considered a key complication of obesity [2], a complex disease characterized by the excessive accumulation of body fat that appears to be a major global health problem. As indicated by the World Health Organization (WHO) and by other reports, chronic obesity develops over a long time and may trigger specific metabolic abnormalities, such as pro-inflammatory conditions, dyslipidemia, hypertension, insulin resistance, glucose intolerance, non-alcoholic fatty liver disease (NAFLD), type 2 diabetes and some types of cancers [3,4,5,6]. Therefore, the prevention and treatment of obesity appear to be a major therapeutic concern worldwide [7]. The main driver of obesity is the imbalance between energy intake and energy expenditure. In particular, obesity is promoted by over-eating and the consumption of nutrient-poor foods enriched with saturated fats and high levels of sugar, accompanied by a sedentary lifestyle [8]. This is further supported by animal studies, where the excessive consumption of a high-fat diet (HFD) is one of the main factors leading to metabolic disorders [9].

The human intestine harbors a complex and dynamic population of microorganisms [10], which is often collectively referred to as the gut microbiota. The current line of evidence emphasizes the close association of gut microbial communities with host health and diseases [11]. Interactions between the gut microbiota and host metabolism are mediated by many factors, including inflammation caused by gut-barrier defects and the metabolism of short-chain fatty acids and bile acid [12]. An imbalance in the gut microbial communities (dysbiosis) is linked to clinical manifestations that are associated with obesity [13] and metabolic disorders, such as type 2 diabetes [14,15]. Several animal studies reported that the consumption of a HFD modulates the gut microbial population. Such changes may enhance intestinal permeability due to gut-barrier disintegration, ultimately leading to the onset and development of metabolic endotoxemia, inflammation and metabolic disorders [16,17,18,19]. Therefore, regulating the gut microbial population via microbiome-based treatments, such as probiotics, prebiotics, fecal microbiota transplantation (FMT), metabolic surgery and drugs, may represent an effective targeted therapeutic strategy for the prevention and treatment of metabolic syndromes [12].

Several lines of evidence suggest that probiotics and their fermented food products have beneficial impacts on health [20]. Specifically, probiotics help maintain the homeostasis of the host gut microbial communities, alleviate immune disorders and offer protection from inflammatory bowel diseases, type 2 diabetes and obesity [21,22,23,24,25,26]. In particular, *Akkermansia muciniphila* (*A. muciniphila*), a mucin-degrading bacterium and one of the most abundant single species in the human intestine (0.5–5% of the total bacteria), is a promising probiotic candidate [27,28,29]. Several studies have indicated the beneficial impact of *A. muciniphila* in maintaining gut health and glucose homeostasis, as well as preventing and ameliorating inflammation, metabolic disorders, obesity and associated complications [26,28,30,31,32,33,34,35]. On the other hand, despite these beneficial effects, the safety profile with the therapeutic use of live probiotics is still a matter of concern. The major risks include the following: systemic infections due to translocation, particularly in vulnerable patients and pediatric populations; acquisition of antibiotic resistance genes; or interference with the gut microbial colonization in infants. To avert these risks, the interest in applying non-viable microorganisms or microbial cell extracts as probiotics, mainly heat-killed or pasteurized probiotic bacteria, has increased. Heat-killed probiotic microbiota retains its key favorable properties and beneficial effects in vitro, animal models and clinical studies [36]. Therefore, these killed or pasteurized bacteria may have more advantages over live probiotics, mainly because of the safety profile.

A recent study reported that the treatment of mice with pasteurized *A. muciniphila* improves HFD-induced metabolic disorders by preventing body weight gain and caloric intake, reducing the total fat level, alleviating liver steatosis, improving glucose homeostasis and insulin sensitivity and restoring the intestinal membrane integrity [26], which is in agreement with previous reports [28,30,31]. On the other hand, emerging evidence indicates that the extent of the effects of probiotics on the host differed between the live and heat-killed forms of the bacteria [37,38]. This prompted the present study to compare the effects of live and pasteurized *A. muciniphila* on obesity, metabolic dysregulation, associated inflammatory insult and gut-barrier integrity using a HFD-induced mouse model.

## 2. Materials and Methods

### 2.1. Preparation of A. muciniphila

The EB-AMDK19 strain of *A. muciniphila* was isolated from healthy Korean subjects in a previous study [26] and the genomic features of this strain were confirmed (accession number, NZ_CP025834). A reference strain of *A. muciniphila* (ATCC BAA-835) used as a control in the experiments was procured from the American Type Culture Collection (Manassas, VA, USA). Luina Bio Pty Ltd. (Darra, Australia) supplied the live biotherapeutic and pasteurized products (heat-inactivated cell) of *A. muciniphila*. Briefly, all *A. muciniphila* strains were grown in a soy-peptone-based medium containing the following (l-1): 20 g of soy-peptone, 10 g of yeast extract, 2.5 g of K_2_HPO_4_, 5 g of N-acetyl-D-glucosamine, 5 g of D-lactose, 2.5 g of D-fructose, 8 g of L-aspartic acid, 0.1 mg of cyanocobalamin and 0.5 g of L-cysteine hydrochloride at 37 °C in an anaerobic chamber filled containing 80% N_2_ and 20% CO_2_. For in situ heat inactivation, the culture broth was heat-inactivated in a fermenter for 30 min at 70 °C with the stirrer speed set to 100 rpm. Subsequently, the broth was centrifuged at 12,000× *g* for 10 min at 4 °C. The bacterial cell pellet was resuspended in sterile PBS buffer to achieve an adequately dispersed solution. Finally, both the live and pasteurized bacterial cells were lyophilized. The live cells and non-viable cells were counted by colony-forming unit count using the spread plate technique and the direct counting method using a Hausser counting chamber, respectively.

### 2.2. Animals, Treatments and Sampling

The animal study was approved by the Institutional Animal Care and Use Committee of Dongguk University (Approval number: IACUC-201911192) and was performed in compliance with the ‘Guide for the Care and Use of Laboratory Animals’. Sixty-three male C57BL6/N mice (6 weeks old, 20 ± 1 g) were procured from Orientbio (Seongnam-si, Gyeonggi-do, Republic of Korea). The normal diet and HFD were purchased from Research Diets Inc. (New Brunswick, NJ, USA). The normal diet was composed of 20% protein, 64% carbohydrate and 7% fat (total calories, 4 kcal/g, 16% calories from fat). In contrast, the HFD was formulated with 26% protein, 26% carbohydrate and 48% fat (total calories, 5.24 kcal/g, 60% calories from fat). The animals were placed into cages (3 animals/cage) and acclimatized for one week at 25 °C in 50–60% humidity and exposure to a 12 h light/12 h dark cycle with access to normal diet and water ad libitum. All mice were weighed and divided randomly into seven groups (*n* = 9), so the differences in average body weights among the groups were minimal. The grouping was performed as follows: (1) Normal, free access to normal diet and water; (2) HFD, free access to HFD and water; (3) Xen, free access to HFD and water and treated with the anti-obesity drug Orlistat (Xen^®^, Roche, Milano, Italy); (4) L.B and (5) P.B, free access to HFD and water and treated with the lyophilized form of live and pasteurized *A. muciniphila* type strain BAA-835, respectively; (6) L.A and (7) P.A, free access to HFD and water and treated with the lyophilized form of live and pasteurized *A. muciniphila* strain 19, respectively. The bacterial samples were administered to the animal groups via oral gavage at 1 × 10^8^ CFU/animal using sterile PBS as a vehicle. Xen^®^ dissolved in PBS was administered to the Xen group orally at a dose of 10 mg/kg. The mice in the normal and HFD groups were administered PBS orally as the vehicle instead of the bacteria or Xen^®^. The treatments with the above-mentioned agents and vehicle were performed five times per week for the 12-week study period. The body weight of the mice was measured weekly and before sacrifice on the last day of the study period as the final body weight.

At the end of the study period, after a 12 h fast, the mice were sacrificed under anesthesia induced by the intraperitoneal administration of Zoletil^®^ (tiletamine-zolazepam, Virbac, Carros, France) and Rompun^®^ (xylazine-hydrochloride, Bayer, Leverkusen, Germany), each at a dose of 1 mL/kg. Blood was collected immediately through a cardiac puncture, as described earlier [39] and subjected to clotting for 30 min at room temperature. The sera were separated by centrifuging the blood samples at 2000× *g* for 15 min and finally stored at −80 °C until further use. The liver, intestine and adipose tissues were excised quickly, washed in ice-cold PBS and blotted and the weights of the liver and adipose tissues were measured. Some portions of the tissues were immediately snap-frozen in liquid nitrogen and stored at −80 °C for further use in gene expression analyses. Other portions of the tissues dedicated to histological analysis were either fixed in a 4% (*w*/*v*) aqueous solution of formaldehyde (for hematoxylin and eosin (H&E) and alcian blue/periodic acid Schiff (AB/PAS)), or stored immediately at −80 °C (for Oil Red O staining).

### 2.3. Analyses of Serum Biochemical Parameters

The serum levels of total cholesterol (TC), triglyceride (TG), glutamic oxaloacetic transaminase (GOT) and glutamic pyruvic transaminase (GPT) were determined using commercial assay kits (Asan Pharmaceutical Co., Seoul, Korea) according to the manufacturer’s instructions. The serum insulin level was measured using an ultrasensitive mouse insulin ELISA kit (MIoBS, Yokohama-shi, Japan).

### 2.4. Oral Glucose Tolerance Test (OGTT)

An OGTT was performed on day 3 of the last week of the study period. Briefly, the 14 h fasting-adapted mice were administered orally with a sterilized aqueous glucose solution (Sigma-Aldrich, St. Louis, MO, USA) at 2 g/kg body weight. Blood was collected from the tail vein of the animals at 0, 30, 60, 90 and 120 min of glucose administration. The blood glucose level was determined using glucose test strips and a handheld blood glucose meter (Accu-Chek Active; Roche Diagnostics GmbH, Mannheim, Germany). The glucose area under the curve (AUC) was extrapolated by plotting the glucose concentration (mmol/L) as a function of time (min) to assess the glucose tolerance.

### 2.5. Histological Analysis

The formaldehyde-fixed liver, intestine and epididymal fat tissues were dehydrated using a series of increasing concentrations of alcohol followed by xylene. The tissues were then embedded in paraffin blocks and sectioned at a 4 µm thickness using a Leica RM2235 rotary microtome (Leica, Nussloch, Germany). For H&E and AB/PAS staining of the desired tissues, the sections were placed on positively charged silicon-coated glass slides, deparaffinized with xylene and then rehydrated using a graded series of decreasing alcohol concentrations. Finally, the liver and epididymal adipose tissue sections were stained with H&E solution (Sigma-Aldrich) and the intestinal sections were stained with an alcian blue/PAS staining kit (Abcam, Cambridge, MA, USA), in accordance with previous reports [40,41]. For Oil Red O staining of the liver, the hepatic tissues were embedded in FSC 22 Frozen Section Compound (mounting medium) (Leica Biosystems, Buffalo Grove, IL, USA) and frozen at −30 °C. The tissues were then sectioned at a 12 μm thickness using a Leica CM1860 Cryostat microtome (Leica Microsystems, Nussloch, Germany). The sections were placed on silicon-coated glass slides as mentioned above and finally stained with an Oil Red O staining solution (O1391, Sigma-Aldrich, St. Louis, MO, USA), as described previously [18].

All stained tissues were examined by optical microscopy (BX61 Olympus, Tokyo, Japan) with the appropriate settings for brightfield/darkfield and fluorescence imaging. The images were captured using a DP70 digital camera (Olympus) at 100×, 200× and 400× magnifications. The histological parameters, such as the diameter and area of the adipocytes in the epididymal fat tissue, as well as the number of goblet cells, crypt depth and mucus layer thickness in the intestinal tissue sections, were analyzed, as described previously [26,41] using ImageJ, a public domain Java-based image-processing software developed at the National Institutes of Health (Bethesda, MD, USA).

### 2.6. Real-Time PCR

The total RNA was extracted from the liver, epididymal fat and intestinal tissues using a TRIsureTM reagent (Bioline Reagent, London, UK) according to the manufacturer’s instructions. The quality and quantity of the isolated RNA were verified by measuring the optical densities at 260 nm and 280 nm using a nanodrop spectrophotometer (Implen, Munich, Germany). cDNA was synthesized by reverse transcription of 1 μg of extracted RNA using an oligo-(dT) 18 primer (Thermo Fisher Scientific, Waltham, MA, USA) and RT PreMix kit (Bioneer, Daejeon, Korea). Real-time PCR was performed on a Light Cycler 480TM device (Roche Applied Science, Basel, Switzerland) in a 96-well plate using a SYBR^®^ Green real-time PCR Master Mix (Toyobo, Tokyo, Japan) and specific primer sets (Supplementary Materials Table S1). For PCR amplification, the following conditions were maintained: an initial denaturation step at 95 °C for 10 min followed by 40 cycles of amplification involving denaturation at 95 °C for 10 s, annealing at 55–58 °C for 5 s and extension at 72 °C for 10 s. The obtained PCR data were processed and analyzed using the dedicated Light Cycler software (version 1.2, Roche Applied Science) and normalized using glyceraldehyde-3-phosphatase dehydrogenase (GAPDH) as the housekeeping gene. The relative expression levels of the genes were measured, as described previously [16,19,26].

### 2.7. Flow Cytometry

The spleen and mesenteric lymph node (MLN) were excised immediately after sacrifice of the animals. After quickly removing the extra connective tissues and fat, each tissue sample was placed into a cell strainer (pore size of 40 μm, SPL Life Sciences, Pochun-si, Gyeonggi-do, Korea) and fitted into a centrifuge tube. The tissue was crushed by applying pressure with the flat end of a plunger taken from a sterile syringe. The plunger and cell strainer were then rinsed thoroughly with 5 mL of PBS, which was collected into the centrifuge tube. The cells were then centrifuged at 300× *g* for 5 min at 4 °C. The resulting cell pellet was resuspended in a cold ammonium–chloride–potassium (ACK) lysing buffer (Gibco, Thermo Fisher Scientific, Waltham, MA, USA) and incubated for 5 min on ice to lyse the red blood cells. Subsequently, the cells were washed with cold PBS and centrifuged at 300× *g* for 5 min at 4 °C. The cell pellet was resuspended in 5 mL of Dulbecco’s phosphate-buffered saline (Sigma-Aldrich St. Louis, MO, USA) containing 0.05% BSA (*w*/*v*) and 2 mM EDTA (PBE buffer) to perform single-cell isolation. The single-cell preparations were then transferred to a 96-well plate (Corning, Corning, NY, USA) at a density of 2 × 10^6^ cells/well in PBE buffer. The cells were stained with near-IR fluorescent reactive dye using a LIVE/DEAD™ Fixable Near-IR Dead Cell Stain Kit (#L10119, Thermo Fisher Scientific, Waltham, MA, USA), according to the manufacturer’s instructions, to distinguish the live cells during the process of gating in subsequent flow cytometry. The cells were then stained for CD4 and CD8 using a mixture of Peridinin Chlorophyll Protein-Cyanine5.5 (PerCP/Cyanine5.5)-labeled anti-mouse CD4 and Phycoerythrin (PE)-conjugated anti-mouse CD8a (#12-0081-83, Thermo Fisher Scientific, Waltham, MA, USA) antibodies. After washing with PBE buffer, the cells were stained for Foxp3 using a Bioscience™ Foxp3/Transcription Factor Staining Buffer Set (#12-0081-83, Thermo Fisher Scientific, Waltham, MA, USA) and an Allophycocyanin (APC)-conjugated monoclonal anti-Foxp3 antibody (#17-5773-80, Thermo Fisher Scientific, Waltham, MA, USA) according to the manufacturer’s protocol. Subsequently, the cells were washed with PBE buffer, resuspended in PBE buffer and assessed using a Becton Dickinson FACSVerse™ flow cytometer (Becton Dickinson Korea, Seoul, Korea). The acquired data were processed and analyzed using FlowJo software 8.0 (Tree Star Inc., San Carlos, CA, USA) to determine the count and population (%) of Treg cells.

### 2.8. Statistical Analyses

All data are expressed as the means ± standard error (SEM). GraphPad Prism Ver 7.04 (GraphPad, San Diego, CA, USA) was used for statistical analyses, which were based on the two-sided unpaired Student’s *t*-test or one-way ANOVA with Bonferroni as a post-hoc test to correct for multiple comparisons unless otherwise indicated. *p*-values < 0.05 were considered significant.

## 3. Results

### 3.1. Effects of A. muciniphila on the Body, Organ and Fat Weights of HFD-Fed Mice

**Figure 1:**
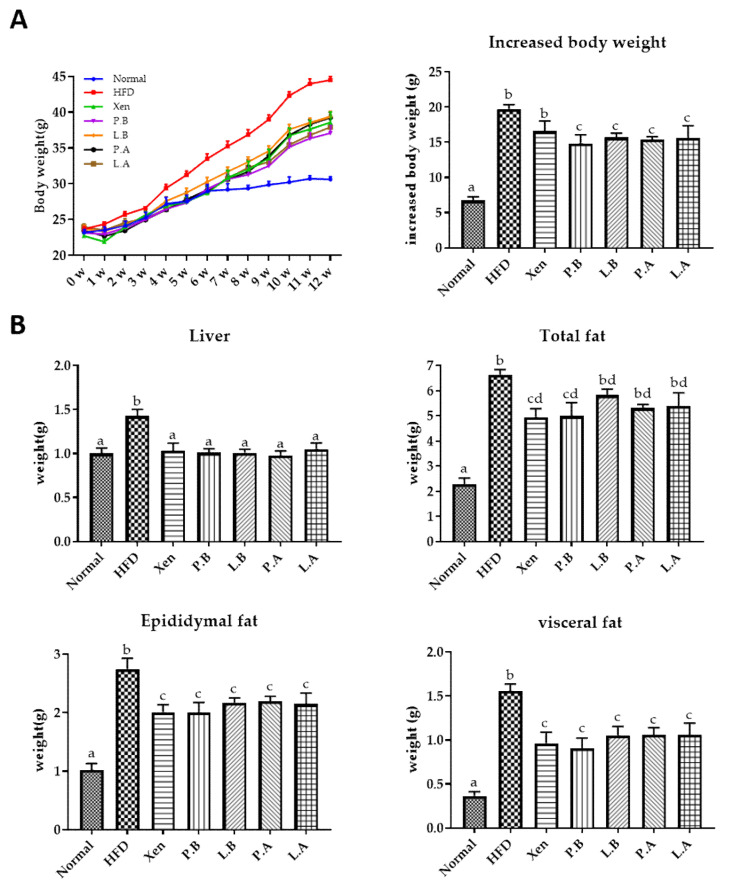
Effects of treatments with *A. muciniphila* or Xen on the (**A**) weekly body weight and body weight gain at week 12 and (**B**) weights of the liver, total fat, epididymal fat and visceral fat at week 12 in the high-fat diet-induced mice. The data are expressed as means ± SD; the differences were statistically evaluated using one-way ANOVA. Means without a common letter significantly differ, *p* < 0.05. HFD, high-fat diet; Xen, orlistat, P.B, pasteurized BAA-835; L.B, live BAA-835; P.A, pasteurized *A. muciniphila*; L.A, live *A. muciniphila*.

At the termination of the study on the 12th week, the following were significantly higher in the HFD group than the normal group: the weekly body weight after one week onwards and the bodyweight gain; liver weight; weights of epidydimal, visceral and total fats; the serum level of TC at week 12. Interestingly, exposure of the HFD-fed animals to P.B, L.B, P.A and L.A, but not Xen, resulted in a significant decrease in body weight gain at week 12 (Figure 1B). Treatment of the HFD group with all the above-mentioned agents for 12 weeks also caused a significant decrease in the weights of the liver, epididymal fat and visceral fat. In contrast, a significant decrease in the total fat and the serum TC level was observed in the HFD-fed mice upon treatment with only Xen and P.B for 12 weeks.

### 3.2. Insulin Sensitivity and Glucose Homeostasis in the HFD Group Were Improved by A. muciniphila

**Figure 2:**
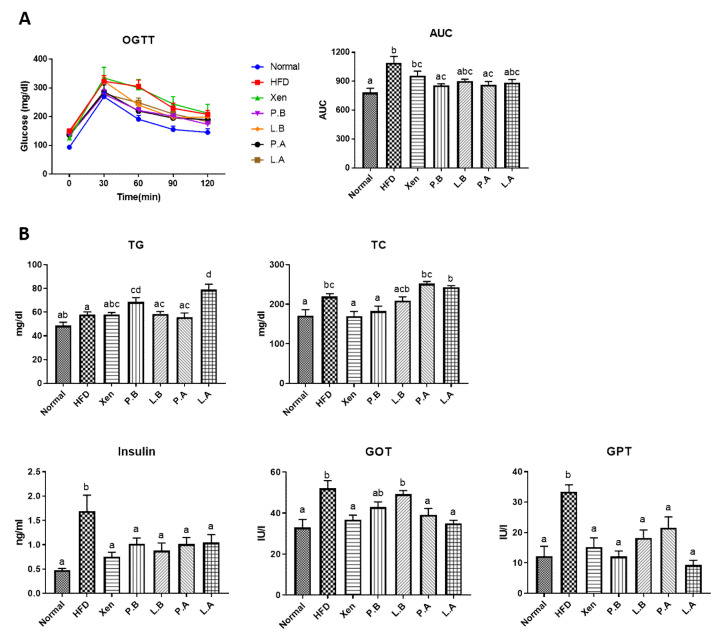
Oral administration of *A. muciniphila* strains improved the obesity parameters in the high-fat diet-induced mice. (**A**) Serum glucose levels at different time points in the oral glucose tolerance test (OGTT). Areas under the curve (AUCs) were constructed, as described in the Materials and Methods Section. (**B**) Serum levels of triglyceride (TG), total cholesterol (TC), insulin, glutamic ox-aloacetic transaminase (GOT) and glutamic pyruvic transaminase (GPT) were assessed to determine the lipid profile, insulin sensitivity, glucose homeostasis and liver function. The data are expressed as means ± SD; the differences were statistically evaluated using one-way ANOVA. Means without a common letter significantly differ, *p* < 0.05. HFD, high-fat diet; Xen, orlistat, P.B, pasteurized BAA-835; L.B, live BAA-835; P.A, pasteurized *A. muciniphila*; L.A, live *A. muciniphila*.

The OGTT results showed that the serum glucose level was significantly higher in the HFD group than the normal group at each time point of measurements (0, 30, 60, 90 and 120 min). On the other hand, the cleaning rate of glucose in HFD-fed mice was improved by a treatment with P.B, L.B, P.A and L.A, but at different degrees (Figure 2A). Our results further revealed that the serum glucose AUC in the HFD-treated mice decreased significantly when exposed to P.B and P.A, but not to other treatments. The consumption of HFD also caused a significant increase in the serum insulin level (Figure 2B) but did not produce any significant changes in the intestinal gene expression of GLP-1 (Figure 5D). In particular, the treatment of HFD-fed mice with all the above-mentioned testing agents caused a significant decrease in the serum insulin level.

### 3.3. Hepatic Fat Accumulation, Steatosis, Inflammation and Liver Injury Were Improved by A. muciniphila in HFD-Induced Mice

**Figure 3:**
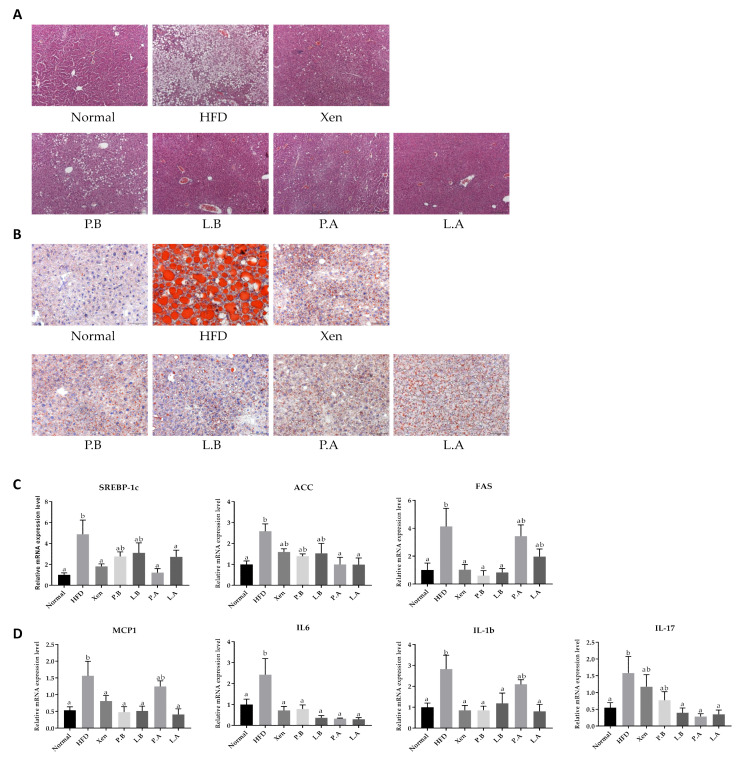
Oral administration of *A. muciniphila* strains suppressed steatosis, lipogenesis and inflammation in the liver of high-fat diet-induced mice. (**A**) Histological analysis of the H&E-stained liver tissue (scale bar, 100 µm). (**B**) Representative Oil Red O staining of the liver tissue to determine the hepatic fat deposition. (scale bar, 100 µm). (**C**) Hepatic gene expressions of lipogenesis markers SREBP-1c, ACC and FAS. (**D**) Hepatic gene expression of proinflammatory chemokine and cytokines. The data are expressed as means ± SD; the differences were statistically evaluated using one-way ANOVA. Means without a common letter significantly differ, *p* < 0.05. HFD, high-fat diet; Xen, orlistat, P.B, pasteurized BAA-835; L.B, live BAA-835; P.A, pasteurized *A. muciniphila*; L.A, live *A. muciniphila*.

As anticipated, the H&E and Oil Red O staining of the liver in the normal group demonstrated a normal histological architecture characterized by the negligible appearance of large vacuoles and minimal lipid deposition (Figure 3A,B). The mice fed a HFD exhibited an aberrant hepatic histological feature dominated by a high abundance of vacuoles associated with marked lipid accumulation, the characteristic features of liver steatosis. This is in keeping with the significantly higher weight of the liver in the HFD group than the normal group (Figure 1B). These findings were also corroborated by the significantly higher hepatic gene expressions of lipogenic markers SREBP-1c, ACC and FAS in the HFD group compared to the normal group (Figure 3C). Furthermore, these results also showed that treatment with a HFD caused inflammatory insult on the liver, as evidenced by the significantly higher hepatic gene expressions of proinflammatory chemokine MCP1 and proinflammatory cytokines IL-1b, IL-6 and IL-17 in the HFD group than the normal group. In keeping with the above-mentioned adverse effects of HFD, the hepatotoxic impact of HFD manifested as significantly higher serum levels of both GOT and GPT in the HFD group than the normal group (Figure 2B).

Treatment of the HFD-fed animals with all the testing agents prevented the hepatic accumulation of lipid droplets and caused marked improvement in the histological profile of the liver, restoring the tissue architecture to almost the normal condition. Consistent with these, the hepatic gene expression of the lipogenic markers in the HFD group was also suppressed by the testing agents, but selectively, as follows: SREBP-1c by Xen, P.A and L.A; ACC by P.A and L.A.; FAS by Xen, P.B and L.B (Figure 3C). The testing agents also showed hepatic anti-inflammatory activity in terms of their abilities to downregulate the gene expression of inflammatory mediators in the liver of HFD-fed animals but at different degrees, as follows: IL-6 by all treatments; MCP1 and IL-1b by all treatments, except P.A; IL-17 by L.B, P.A and L.A. The testing agents had a protective effect against the HFD-induced hepatotoxicity in mice, as evident by a decline in the serum levels of GOT and GPT by the agents, but at different degrees (Figure 2B). In particular, in HFD-fed animals, the serum GPT level was reduced significantly by treatment with all the agents, while the serum GOT concentration was decreased significantly upon exposure to Xen, P.A and L.A, but not to P.B and L.B

### 3.4. A. muciniphila Suppressed the Adipogenesis and Inflammation in Adipose Tissue

**Figure 4:**
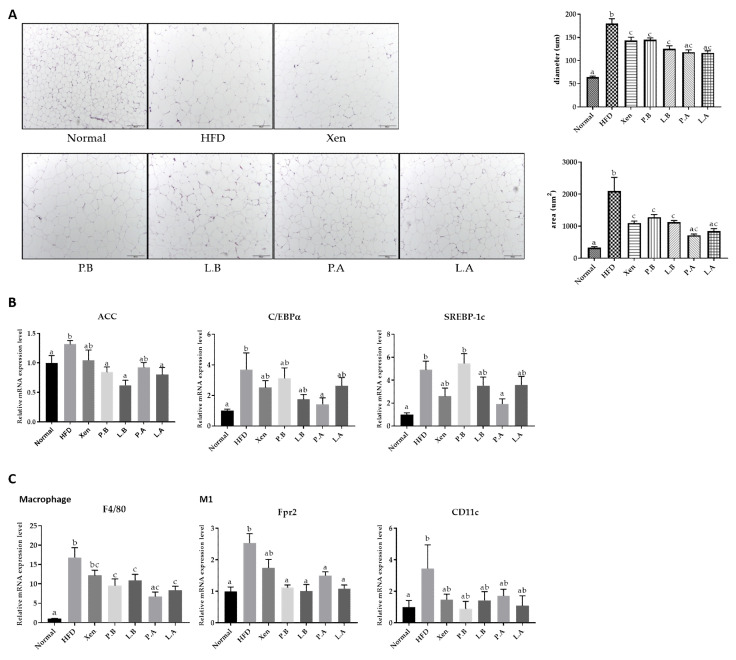
Effect of bacteria on the adipose tissue in the HFD mice. (**A**) Histological analysis (H&E staining) on epididymal fat tissue sections (scale bar, 100 μm). (**B**) Representative mRNA expression levels of the ACC, C/EBPα and SREBP-1c as lipid metabolism markers. (**C**) Percentage expression of macrophages in the epididymal fat tissue. The data are expressed as means ± SD; the differences were statistically evaluated using one-way ANOVA. Means without a common letter significantly differ, *p* < 0.05. HFD, high-fat diet; Xen, orlistat, P.B, pasteurized BAA-835; L.B, live BAA-835; P.A, pasteurized *A. muciniphila*; L.A, live *A. muciniphila*.

H&E staining of epididymal adipose tissue showed that the average diameter and area of the adipocytes were significantly higher in the HFD group than the normal group, indicating marked fat deposition in the HFD-fed animals (Figure 4A). This finding is in keeping with the significantly higher gene expression of lipogenic markers, ACC, C/EBPα and SREBP-1c, in the epididymal fat tissue of the HFD group than the normal group (Figure 4B). Furthermore, exposure to HFD manifested as an inflammatory insult on the fat tissue. This is evidenced by the significantly higher adipose tissue gene expression of universal murine macrophage marker F4/80 and the M1 macrophage markers Fpr2 [42] and CD11c in the HFD group vs. the normal group (Figure 4C).

Exposure of the HFD-fed animals to all the testing agents caused a significant decline in the mean diameter and the area of the adipocytes. Furthermore, the adipose tissue gene expression of lipogenic markers in the HFD group was also decreased significantly by the testing agents, but differently, as follows: ACC by P.B, L.B and L.A; C/EBPα and SREBP-1c by P.A. The testing agents also revealed the anti-inflammatory activities in the adipose tissue of the HFD-fed animals as their treatments suppressed the gene expression of the macrophage markers F4/80 and Fpr2 by P.B, L.B, P.A and L.A, but not by Xen.

### 3.5. A. muciniphila Improved the Intestinal Structure, Inflammation and Barrier Integrity in the HFD-Induced Mice

**Figure 5:**
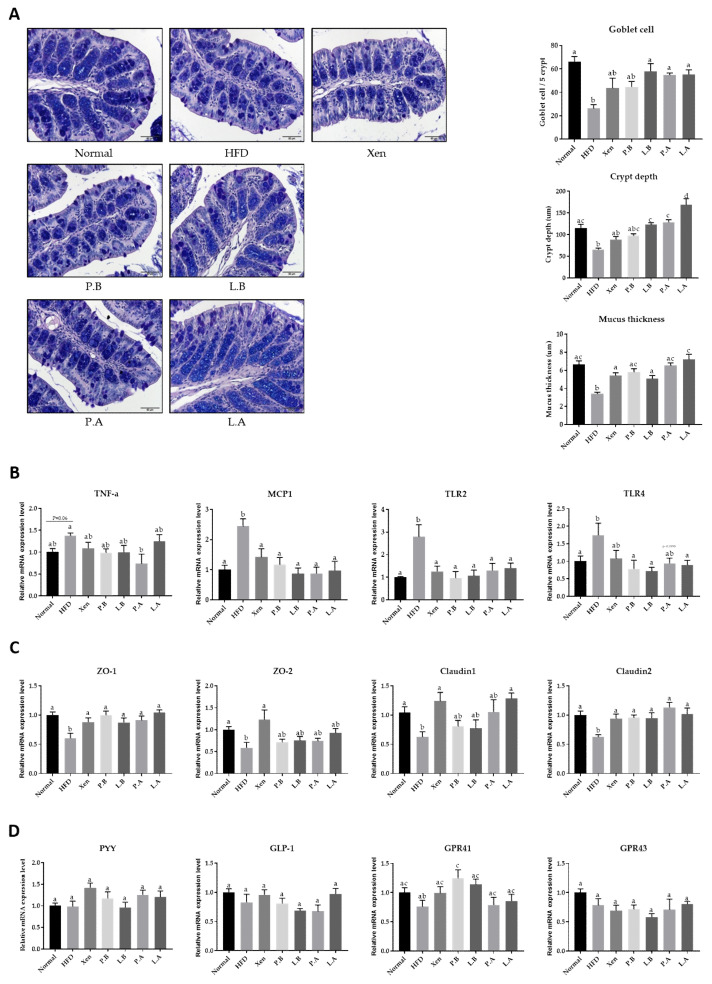
Administration of bacteria improved the intestinal structure, permeability and inflammation, but did not significantly change the levels of gene expression of appetite-regulating factors. (**A**) Histological analysis (AB-PAS staining) of the colonic tissue sections (magnification of 200×). (**B**) mRNA levels of TNF-a, MCP1, TLR2 and TLR4 as inflammation markers. (**C**) Representative gene expressions of ZO-1, ZO-2, Claudin1 and Claudin2 as permeability markers. (**D**) mRNA levels of the appetite markers PYY, GLP-1, GPR41 and GPR43. The data are expressed as means ± SD; the differences were statistically evaluated using one-way ANOVA. Means without a common letter significantly differ, *p* < 0.05. HFD, high-fat diet; Xen, orlistat, P.B, pasteurized BAA-835; L.B, live BAA-835; P.A, pasteurized *A. muciniphila*; L.A, live *A. muciniphila*.

The histological evaluation of the AB/PAS-stained colonic tissue was performed to examine the impact of the testing agents on the intestinal structure of the HFD-fed mice. Consumption of HFD caused a marked disruption in the histological architecture of the intestinal tissue in the animals. In particular, the crypt depth and count of goblet cells in the epithelium displaying positive staining with AB/PAS were reduced significantly in response to the HFD treatment (Figure 5A). Furthermore, a significant decrease in mucus layer thickness was observed in the HFD group compared to the normal group. The results showed that treatment with HFD imposed an inflammatory insult on the intestine, as evidenced by the significant increase in the colonic gene expression of the inflammatory marker MCP1 (Figure 5B). On the other hand, although the change was insignificant, the intestinal mRNA level of TNF-α was increased markedly (*p* = 0.06) in response to treatment with HFD. The toll-like receptor (TLR) family members play an essential role in innate immunity and inflammatory response. Significantly higher intestinal levels of TLR2 and TLR4 gene expression were found in the HFD group than the normal group. The intestinal levels of ZO-1, ZO-2, claudin 1 and claudin 2 gene expression, which are vital epithelial tight junction (TJ) proteins that help maintain the gut-barrier integrity, were significantly lower in the HFD group than the normal group (Figure 5C). On the other hand, no significant differences were observed in the intestinal gene expression of the gut hormone PYY, a peptide that has anorectic effects and reduces body weight, between the normal and HFD groups (Figure 5D). The G protein-coupled receptors (GPCRs) GPR41 and GPR43 play essential roles in mediating the interaction between the host and the gut microbiota, as well as functioning as appetite regulator. No significant changes in the intestinal gene expression of these two proteins were observed in the animals in response to the HFD.

These results showed that the test agents had appreciable protective activities against intestinal inflammation and structural aberration in the HFD-fed mice but to different extents. Exposure of the HFD-treated mice to L.B, P.A and L.A, but not Xen or P.B, resulted in a significant increase in the goblet cell count and crypt depth. Treatment of the HFD-treated animals with all the testing agents caused a significant increase in the mucus layer thickness and the intestinal gene expression of ZO-1 and claudin 2 and a significant decrease in the colonic expression of MCP1 and TLR2 genes. The significant effects of these testing agents on the gene expression of the other intestinal proteins in the HFD group were as follows: downregulation of TNF-α by P.A; suppression of TLR4 by P.B, L.B and L.A; increase in ZO-2 by Xen; upregulation of claudin 1 by Xen and L.A.

### 3.6. A. muciniphila Reduced the Population of Splenic Treg Cells in the HFD Group

**Figure 6:**
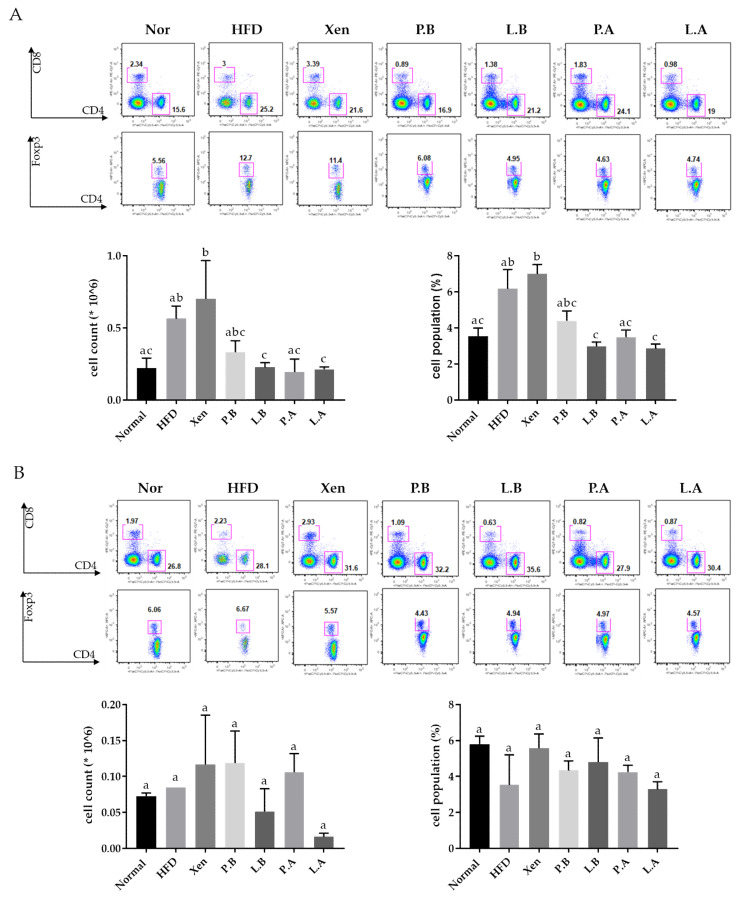
Treg cells numbers and population in the spleen (**A**) and MLN (**B**) tissue. The data are expressed as means ± SD; the differences were statistically evaluated using one-way ANOVA. Means without a common letter significantly differ, *p* < 0.05. HFD, high-fat diet; Xen, orlistat, P.B, pasteurized BAA-835; L.B, live BAA-835; P.A, pasteurized *A. muciniphila*; L.A, live *A. muciniphila*.

This study revealed an insignificant yet definite increase in the number and population (%) of Treg cells (CD4+ Foxp3+) in the spleen of mice in response to the HFD treatment (Figure 6A). On the other hand, exposure of the HFD-fed animals to L.B, P.A, and L.A and L.B and L.A decreased the number and population of the splenic Treg cells significantly, respectively (Figure 6A). In the MLN tissue, however, the number and population of Treg cells were similar in the normal and the HFD groups. In particular, treatment of the HFD-fed animals with L.A caused a significant decrease in the Treg cell count in MLN. Except for that, the testing agents had no other significant impact on the Treg cell number and population in the MLN of the HFD group (Figure 6B).

## 4. Discussion

In the present study, we compare the beneficial effects of live and pasteurized *A. muciniphila* against metabolic disorder and associated inflammation, as well as gut-barrier function, using HFD-fed mice as a model. Consumption of a HFD triggers the onset and development of obesity, which is manifested by an increase in body weight, total fat, visceral fat and subcutaneous fat [43], in keeping with our results. We also observed significantly higher levels of adipose tissue gene expression of the lipogenic markers, ACC, C/EBPα and SREBP-1c in the HFD group than in the normal group. The transcription factor C/EBPα, which plays a vital role in regulating adipogenesis in coordination with PPAR-γ, triggers the gene expression of several adipogenesis/lipogenesis activators and enzymes. Such events lead to the synthesis of fatty acids and triglycerides, which ultimately facilitate adipocyte differentiation [44,45]. SREBP-1c primarily regulates the genes involved in de novo lipogenesis and triglyceride synthesis, including ACC, which plays an essential role in modulating fatty acid synthesis and degradation [46,47]. In particular, treatment of HFD-fed mice with both *A. muciniphila* strains, either alive or pasteurized, resulted in significant decreases in body weight gain and reduced epididymal and visceral fat weights. In contrast, exposure of the HFD-induced animals to P.B, but not other *A. muciniphila* preparations, caused a significant decline in the total fat and the serum TC level. Furthermore, treatment of the HFD-fed animals to both *A. muciniphila* strains, irrespective of their viability, resulted in a significant decrease in the mean diameter and the area of the adipocytes in epididymal fat tissue. In parallel, the adipose tissue gene expression of the lipogenic markers in the HFD group was also decreased significantly by the *A. muciniphila* strains, but in a differential manner. Overall, our results support the anti-obesity, anti-adipogenic and anti-lipogenic activities of both *A. muciniphila* strains, which is in agreement with an earlier report [26]. Accumulating evidence suggests that, in animals, in addition to obesity, HFD consumption induces liver damage, which is similar to the phenotype evident in humans suffering from NAFLD [48]. In the present study, in parallel with the increase in body weight, the liver weight of mice was also augmented by the consumption of HFD. The accumulation of lipids in the liver and insulin resistance are significant factors contributing to hepatic steatosis development [49]. In the present study, the histological evaluation of H&E- and Oil Red O-stained liver tissues from the HFD-fed animals revealed typical hepatic steatosis characterized by markedly higher lipid deposition. Furthermore, the expression levels of the hepatic genes, SREBP-1c, ACC and FAS, a vital enzyme that regulates the de novo biosynthesis of long-chain fatty acids [50], were significantly higher in the HFD group than in the normal group. These observed events were also associated with liver damage because the serum levels of GOT and GPT, vital markers of liver injury, were elevated significantly in the animals in response to HFD intake. In particular, exposure of the HFD-fed animals to both *A. muciniphila* strains, either alive or pasteurized, strongly blocked the hepatic deposition of lipid droplets, resulting in marked improvements in the liver histological features, almost restoring the tissue structure to the normal state. Significant downregulation of the hepatic gene expression of the lipogenic markers in the HFD group by the *A. muciniphila* strains was also observed as follows: SREBP-1c and ACC by P.A and L.A; FAS by P.B and L.B. Overall, these findings support the hepatoprotective activities of both *A. muciniphila* strains against HFD-induced steatosis, which is in agreement with an earlier report [26]. 

Increasing evidence suggests that insulin resistance is associated with obesity, particularly visceral adipose tissue accumulation [51]. In the present study, the peak OGTT glucose level, OGTT AUC and serum insulin concentration were significantly higher in the HFD-induced obese group than the normal group, indicating insulin resistance, impaired glucose tolerance and homeostasis in the latter group. Treatment of the HFD-fed mice with both *A. muciniphila* strains in pasteurized form but not in the live form decreased the OGTT AUC significantly. In contrast, exposure of the HFD-fed animals to both *A. muciniphila* strains, either alive or pasteurized, decreased the serum insulin level significantly. Based on these observations, it is conceivable that, in the HFD group, although both the live and pasteurized forms of the *A. muciniphila* strains have a beneficial impact on glucose homeostasis, the latter form of the bacteria was more potent than the former in improving the glucose tolerance.

Although the overweight condition and obesity are generally considered the outcome of an imbalance in energy homeostasis, growing lines of evidence suggest that metabolic disorder is associated with alterations in immunity, such as chronic low-grade inflammation [52]. The consumption of a HFD elevates the serum LPS level, leading to endotoxemia [53]. Several lines of evidence also suggest that HFD could induce the production of proinflammatory cytokines, including IL-1b, IL-6, TNF-α, proinflammatory chemokine MCP1 and toll-like receptors (TLR2 and TLR4), in various tissues, including the liver and colon. Such events ultimately trigger low-grade inflammation that may be associated with obesity, insulin resistance and other metabolic disorders [49,54,55]. Furthermore, macrophage accumulation in adipose tissue is a typical hallmark of obesity [56]. In particular, diet-induced obesity is associated with an increase in the M1 macrophage population in the adipose tissue [53]. These macrophages secrete TNF-α, IL-6, IL-1b and MCP1, which induce insulin resistance [57].

In keeping with the above reports, the present study demonstrated significantly higher hepatic gene expression of IL-1b, IL-6, IL-17 and MCP1 in the HFD group than the normal group, while the intestinal gene expression of MCP1, TLR2 and TLR4 was significantly higher in the HFD group than in the normal group. In the adipose tissue, the gene expression of Fpr2 and CD11c, as indirect measures of the M1 macrophage population, was significantly higher in the HFD group than in the normal group. A previous study showed that the gene expression of Fpr2 was up-regulated over 100-fold during differentiation of M1 macrophages but down-regulated during differentiation of M2 macrophages, supporting Fpr2 as an M1 macrophage-specific marker [42]. Indeed, Fpr2 has been shown to play an important role in M1 macrophages as its deficiency triggers an M2 phenotype shift [58]. Treatment of the HFD-fed animals with both strains of *A. muciniphila*, either alive or pasteurized, significantly decreased the gene expressions of hepatic IL6, intestinal MCP1 and TLR2 and adipose tissue Fpr2. Exposure of the HFD group to all preparations of *A. muciniphila*, except P.A, significantly downregulated the gene expression of hepatic MCP1 and intestinal TLR4. In addition, a significant decrease in hepatic IL-17 and intestinal TNF-α gene expression was observed in the HFD group in response to the treatments with L.B, P.A and L.A., and P.A, respectively. Overall, our findings support the strong protective activities of both *A. muciniphila* strains against HFD-induced inflammatory insults on different organs, which agrees with an earlier report [26].

The anti-inflammatory actions of the *A. muciniphila* strains prompted an evaluation of the protective activity of these bacterial strains against a HFD-induced insult on the intestine. Several lines of evidence suggest that in diet-induced obese mice, the anti-inflammatory process is associated with a lowering of the intestinal permeability by ameliorating the gut-barrier integrity [59,60]. Many studies showed that the epithelial tight junction proteins, including ZOs, occludin and claudins, are the crucial players controlling the intestinal-barrier function and regulating intestinal permeability [61]. In the present study, an aberrant histological architecture was observed in the AB/PAS-stained intestinal tissue of the HFD-fed mice. In particular, the crypt depth, count of the epithelial goblet cells and mucus layer thickness were lower in the HFD group than the normal group, which is similar to previous reports [26,62,63]. Furthermore, HFD treatment impairs the gut-barrier function, which is associated with the decreased intestinal expression of ZO-1, occludin and claudin 1 [17,64]. In the present study, intestinal gene expression of ZO-1, ZO-2, claudin 1 and claudin 2, was significantly downregulated in the animals in response to HFD consumption. Treatment of the HFD-treated mice with L.B, P.A and L.A, but not P.B, resulted in a significant increase in the goblet cell count and crypt depth. Conversely, exposure of HFD-fed animals to both strains of *A. muciniphila*, either alive or pasteurized, promoted a significant increase in the mucus layer thickness and the intestinal expression of ZO-1 and claudin 2, while the intestinal gene expression of claudin 1 was upregulated significantly in the HFD group upon treatment with only L.A. Overall, these results support the appreciable intestinal protective effects of both *A. muciniphila* strains on the HFD-fed animals, in keeping with a previous study [26].

Treg cells, which play a vital role in maintaining the immune homeostasis, are a subset of CD4+ T cells characterized by their expression of the transcription factor forkhead box protein P3 (Foxp3). These cells serve as critical negative regulators of inflammation and produce anti-inflammatory cytokines, such as IL-10, IL-35 and TGF-β [65]. In the present study, HFD-induced inflammatory insults to the various tissues of the mice, including the intestine, were accompanied by an insignificant yet definite increase in both the number and population (%) of Treg cells (CD4+ Foxp3+) in the spleen. This is in keeping with a recent report showing an increased presence of Tregs in children diagnosed with IBD [66], a chronic inflammatory state of the intestine. Such an event, which also showed a positive correlation with the inflammatory marker c-reactive protein, was explained as a probable compensative reaction to tissue inflammation [66]. In our study, an exposure of the HFD-fed animals to specific *A. muciniphila* preparations with either live or pasteurized bacteria significantly decreased the number and population of the splenic Treg cells, supporting the role of these bacteria in reducing the inflammatory insults.

## 5. Conclusions

Overall, our findings clearly show that with the use of the pasteurized form, there was no loss of therapeutic potency of *A. muciniphila*, compared to its live form. Furthermore, in gross, the efficiency of this probiotic against obesity and metabolic dysregulation was superior to that of Orlistat.

## Supplementary Materials

The following are available online at https://www.mdpi.com/article/10.3390/microorganisms9102039/s1. Table S1. Sequences of the primer used in quantitative real-time PCR.
