# Supplementary material for: Effects of Live and Pasteurized Forms of Akkermansia from the Human Gut on Obesity and Metabolic Dysregulation
Source: Microorganisms. 2021 Sep 27;9(10):2039. doi: 10.3390/microorganisms9102039 (PMC8538271; doi:10.3390/microorganisms9102039)
Supplement: Supplementary file 1 [file microorganisms-09-02039-s001.zip › microorganisms-1331608-supplementary.pdf]

microorganisms

MDPI

Supplementary

Table S1. Sequences of the primer used in quantitative real-time PCR.

**SREBP-1c**	**Forward**	**GGAGCCATGGATTGCACATT**
	**Reverse**	**GGCCCGGGAAGTCACTGT**
ACC	Forward	CCTCCGTCAGCTCAGATACA
	Reverse	TTTACTAGGTGCAAGCCAGACA
FAS	Forward	AGGGGTCGACCTGGTCCTCA
	Reverse	GCCATGCCCAGAGGGTGGTT
MCP1	Forward	AAGAGATCAGGGAGTTTGCT
	Reverse	CTGCCTCCATCAACCACTTT
IL6	Forward	CCTCTGGTCTTCTGGAGTACC
	Reverse	ACTCCTTCTGTGACTCCAGC
IL-1b	Forward	TGCCACCTTTTGACAGTGATG
	Reverse	AAGGTCCACGGGAAAGACAC
IL-17	Forward	TCAGCGTGTCCAAACACTGAG
	Reverse	CGCCAAGGGAGTTAAAGACTT
C/EBPα	Forward	AGCAACGAGTACCGGGTACG
	Reverse	TGTTTGGCTTTATCTCGGCTC
F4/80	Forward	CGTCAGGTACGGGATGAATATAAG
	Reverse	CTATGCCATCCACTTCCAAGAT
Fpr2	Forward	TTACATCTACCACAATGTGAACTA
	Reverse	CAGCGACTCTGAAGAAGAGCAAG
CD11c	Forward	GTGCCCATCAGTTCCTTACA
	Reverse	GAGAAGAACTGTGGAGCTGAC
cMyc	Forward	CAGCGACTCTGAAGAAGAGCAAG
	Reverse	GGGTTTGCCTCTTCTCCACAG
CD206	Forward	GGAATCAAGGGCACAGAGTTA
	Reverse	ATTGTGGAGCAGATGGAA
CD163	Forward	CAGACTGGTTGGAGGAGAAATC
	Reverse	TGACTTGTCTCTGGAAGCTG
TNF-α	Forward	GACCCTCACACTCAGATCATCTTCT
	Reverse	CCACTTGGTGGTTTGCTACGA
TLR2	Forward	AAGGAGGTGCGGACTGTTTC
	Reverse	GAGCCAAAGAGCTCGTAGC
TLR4	Forward	CCTGATGACATTCCTTCTTCAAC
	Reverse	TTGTTTCAATTTCACACCTGGATAAA
ZO-1	Forward	TTTTTGACAGGGGGAGTGG
	Reverse	TGCTGCAGAGGTCAAAGTTCAAG
ZO-2	Forward	ATGGGAGCAGTACACCGTGA
	Reverse	TGACCACCCTGTCATTTTCTTG
Claudin1	Forward	ACTCCTTGCTGAATCTGAACAGT
	Reverse	GGACACAAAGATTGCGATCAG
Claudin2	Forward	TGAACACGGACCACTGAAAG
	Reverse	TTAGCAGGAAGCTGGGTCAG
PYY	Forward	CGGCAGCGGTATGGAAAAA
	Reverse	TGTGAAGAGCAGTTTGGAGAACA

GLP-1	Forward	GGCACATTCACCAGCGACTAC
	Reverse	CAATGGCGACTTCTTCTGGG
GPR41	Forward	GGGGTCGATACAAGAGT
	Reverse	CTGGCGGAGCTACGTGCT
GPR43	Forward	ACCATCGTCATCATCGTTCA
	Reverse	ACGAAGCGCCAATAACAGAA
